# Mosaic *DICER1* RNase IIIb hotspot mutation with multiple tumors: case report and literature review

**DOI:** 10.3389/fonc.2026.1777961

**Published:** 2026-04-27

**Authors:** Peiyi Yang, Mei Jin, Wen Zhao, Chao Duan, Jie Yu, Yanchao Qu, Libing Fu, Tong Yu, Xiaoman Wang, Ruolan Guo, Chunying Cui, Dong Mei, Yan Su

**Affiliations:** 1 Medical Oncology Department, Pediatric Oncology Center; National Key Clinical Discipline of Pediatric Oncology, Laboratory for Clinical Medicine, Capital Medical University, Key Laboratory of Major Diseases in Children, Ministry of Education, Beijing Children’s Hospital, Capital Medical University, National Center for Children’s Health, Beijing, China; 2 Department of Thoracic Surgery, Beijing Children’s Hospital, Capital Medical University, National Center for Children’s Health, Beijing, China; 3 Department of Urology, Beijing Children’s Hospital, Capital Medical University, National Center for Children’s Health, Beijing, China; 4 Department of Pathology, Beijing Children’s Hospital, Capital Medical University, National Center for Children’s Health, Beijing, China; 5 Imaging Center, Beijing Children’s Hospital, Capital Medical University, National Center for Children’s Health, Beijing, China; 6 Department of Ultrasound, Beijing Children’s Hospital, Capital Medical University, National Center for Children’s Health, Beijing, China; 7 Genetics and Birth Defects Control Center, Beijing Children’s Hospital, Capital Medical University, National Center for Children’s Health, Beijing, China; 8 School of Pharmaceutical Sciences, Capital Medical University, Beijing, China; 9 Department of Pharmacy, Beijing Children’s Hospital, Capital Medical University, National Center for Children’s Health, Beijing, China

**Keywords:** case report, *DICER1*, hotpot mutation, mosaic, multiple tumors

## Abstract

We present a case of a 6-month-old boy who was diagnosed with Peutz–Jeghers polyps, intestine fusiform mesenchymal tumor, Wilms tumor, and Ir type pleuropulmonary blastoma (PPB) successively, with mosaic *DICER1* RNase IIIb hotspot mutation: c.5113G>A p.E1705K. The patient underwent surgeries to resect the intestine polyps, intestine tumor, Wilms tumor, nephroblastomatosis, and PPB, combined with chemotherapy aimed at Wilms tumor and PPB. Diseases were stable at follow-up 27 months since the initial diagnosis.

## 1 Introduction

The *DICER1* gene is located on chromosome 14q32.13 and plays a crucial role in the control of protein translation. Its product, i.e., dicer protein, is a ribonuclease (RNase) III endoribonuclease that is essential for the production of microRNAs (miRNAs), which are formed by the cleavage of pre-miRNA or double-stranded RNA (1). *DICER1*-related tumor predisposition is a cancer-predisposing disorder caused by pathogenic variants in the *DICER1* gene, which are associated with lifetime risks of a variety of neoplastic and dysplastic lesions. Research has revealed that mosaicism for RNase IIIb domain hotspot mutations defines a special category of *DICER1*-related tumor predisposition, clinically distinguished from those with germline or mosaic loss-of-function (LOF) mutations by an earlier onset and the numerous discrete foci of neoplastic disease involving multiple syndromic organ sites (2). We report on a case of mosaic *DICER1* RNase IIIb hotspot mutation with multiple tumors and review the literature.

## 2 Case report

### 2.1 Clinical examination

**Figure 1:**
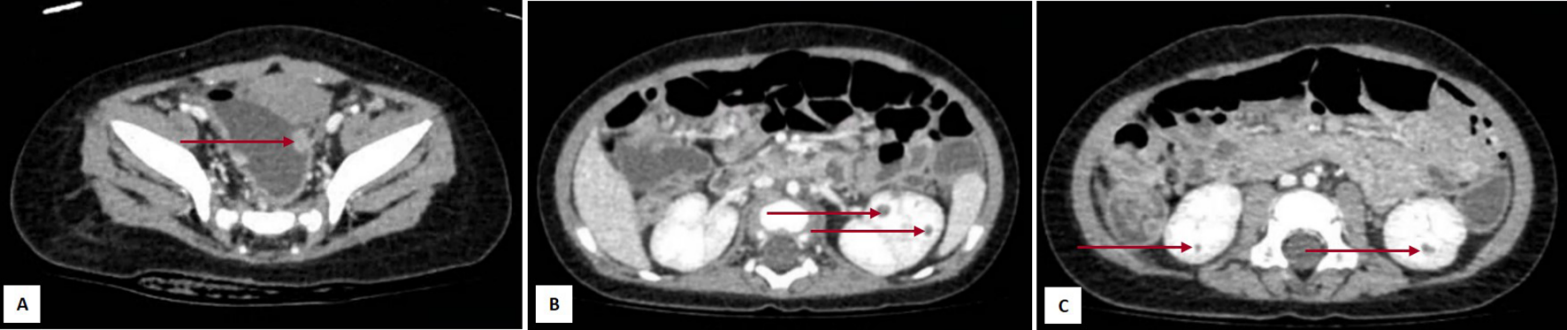
Images of the abdomen CT scan at diagnosis. **(A)** Polyp in the sigmoid. (**B**, **C**) Nodules in both kidneys. The red arrow in Panel **(A)** points to a polyp in the sigmoid colon; the red arrows in Panels **(B, C)** point to nodules in both kidneys.

A 6-month-old boy presented with intermittent vomiting for 7 weeks and seven times of intussusception. For the past seven times, ultrasound at the local hospital all showed intussusception, which was remitted by clyster. X-ray of the chest was normal. At admission to our hospital, ultrasound of the digestive tract showed multiple polyps in the small intestine, with loose intussusception, and another polyp in the sigmoid, 1.0 cm × 0.6 cm × 0.8 cm in size. CT scan also showed multiple nodules of varying sizes from the jejunum to the sigmoid and scattered low-density shadows in both kidneys, with a diameter of approximately 3 mm (**Figure 1**). He underwent the first surgery, i.e., resection of the intestinal tumors. The patient had no signs of developmental delay or overgrowth, with a normal head circumference and facial features.

### 2.2 Histopathology of the first surgery

**Figure 2:**
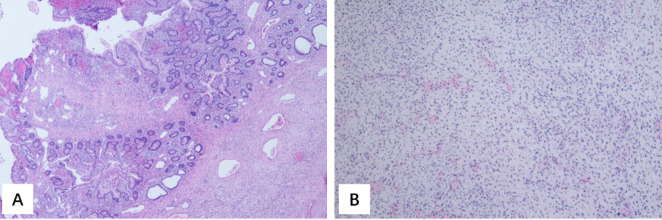
Hematoxylin–eosin (HE) staining of the nodules of the intestinal tract. **(A)** Polyps (×4). **(B)** Fusiform mesenchymal tumor (×10).

Seven nodules showed similar appearance, i.e., dusty pink, like granules, with a diameter of 0.8–1.8 cm. Focal superficial ulceration was identified, accompanied by the proliferation of inflammatory granulation tissue. Pathological consultations across multiple hospitals confirmed a diagnosis of Peutz–Jeghers (P-J) polyps. The other three nodules, 20 cm from the ileocecal junction, were myxoid and smooth, and macroscopic examination showed spindle cell tumor with morphologic features suggestive of sarcoma with *BCOR* alterations (**Figure 2**). However, fluorescence *in situ* hybridization (FISH) showed that there was no break or translocation of *BCOR*. Subsequently, the specimen was sent for pathological consultation at three hospitals. One hospital diagnosed it as a low-grade malignant spindle cell mesenchymal tumor without further specific classification. The other two hospitals favored a diagnosis of congenital infantile fibrosarcoma.

Immunohistochemistry yielded positive results for catenin, CCNB3, cyclin D1, FLI-1, SATB2, TLE1, and MDM2 and negative results for CD117, SMA, S-100, pan-TRK, CD31, BCOR, desmin, myogenin, CD99, and WT1. The Ki-67 index was 50%.

The patient was regularly followed up after the surgery.

### 2.3 Follow-up after the first surgery

**Figure 3:**
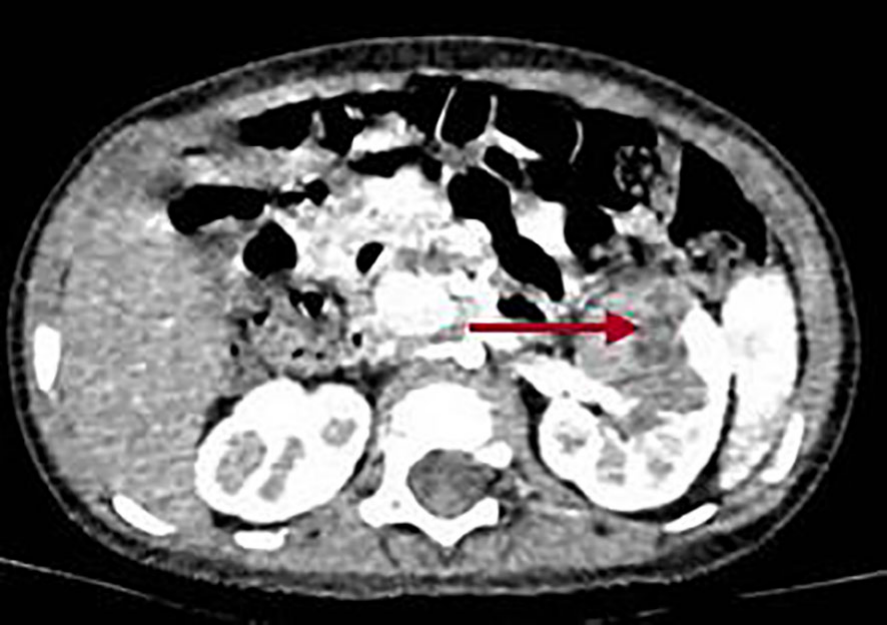
Image of the CT scan 5 months after the first surgery. The arrow points to the lesion in the left kidney.

At the regular assessment 3 and 5 months after the first surgery, the lesions in the left kidney gradually was found to have increased in size, with the largest one measuring 2.8 cm × 2.3 cm × 2.9 cm in the CT scan 5 months after surgery (**Figure 3**). The second surgery then proceeded, resecting the largest mass in the left kidney.

### 2.4 Histopathology of the second surgery

**Figure 4:**
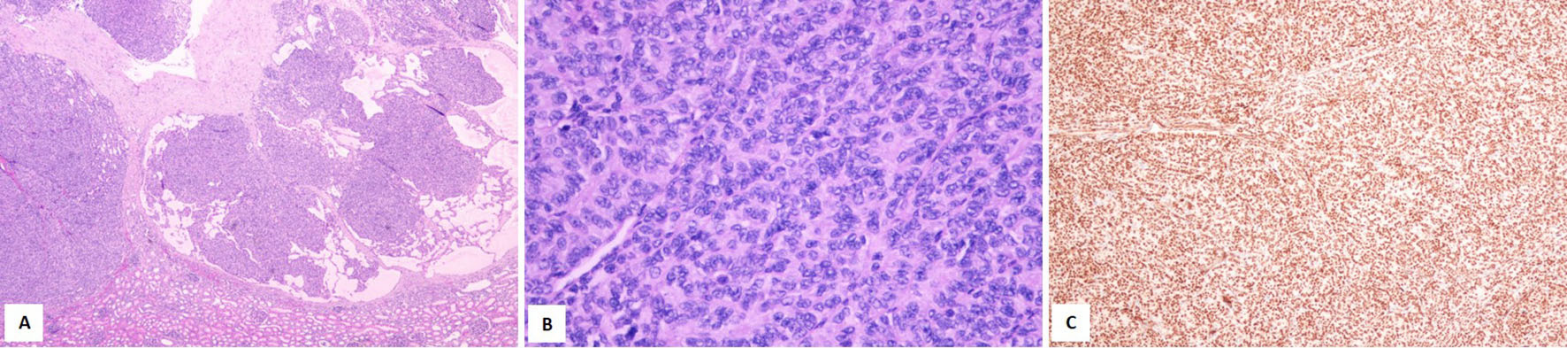
**(A)** Hematoxylin–eosin (HE) staining of the tumor in the kidney (×4). **(B)** HE staining of the tumor in the kidney (×40). **(C)** WT1 staining of the tumor in the kidney showed positive.

The mass weighed 33 g and measured 3.5 cm × 2.6 cm × 1.9 cm. It was solid and off-white. Macroscopic examination revealed the tumor to be lobulated, with a large nucleus; mitotic figures were common, and cell atypia was obvious (**Figure 4**). Immunohistochemistry yielded positive results for WT1, CD56, INI-1, cyclin D1, cytokeratin (CK) (AE1/AE3), desmin, and pan-TRK and negative results for BCOR, EMA, myogenin, S-100, SMA, CD34, TTF1, CgA, Syn, HMB45, CD57, CK7, AMACR, and TFE3. The Ki-67 index was 80%. A diagnosis of Wilms tumor was made.

### 2.5 Treatment after the second surgery

**Figure 5:**
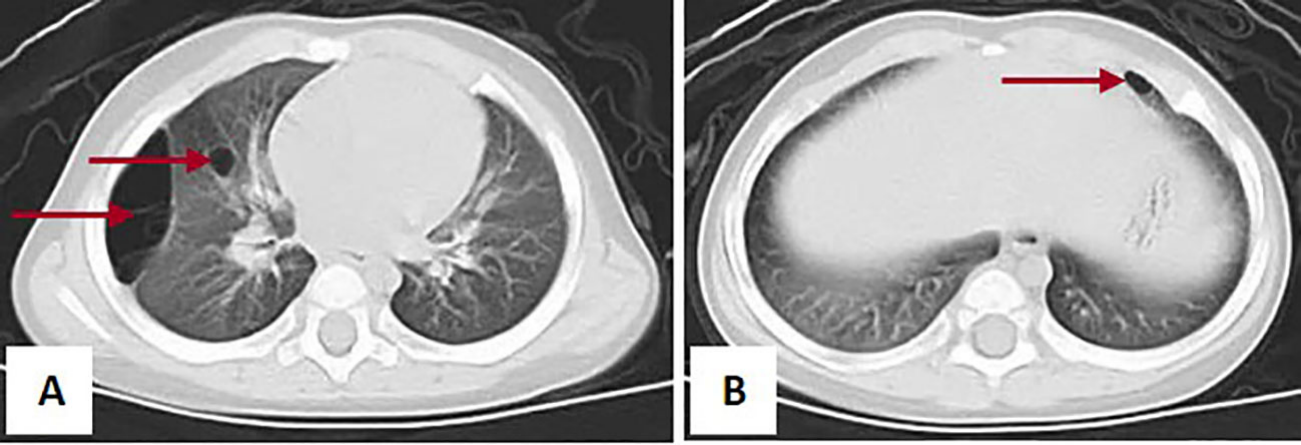
Images of the chest CT scan showed lesions in both lungs. **(A)** Right lung. **(B)** Left lung. The red arrow in Panel **(A)** points to the lesion in the right lung, and the red arrow in Panel **(B)** points to the lesion in the left lung.

Subsequently, the patient was started on chemotherapy with vincristine and actinomycin D (VA). Concurrently, routine chest CT showed multiple cystic lesions in the lungs, which were suspected to be pleuropulmonary blastoma (PPB), considering the young age and history of intestine polyps and Wilms tumor (**Figure 5**). No definite abnormalities were found on ultrasonography of the thyroid gland, mediastinum, pelvic cavity, axillae, inguinal regions, and other sites, as well as on cranial magnetic resonance imaging (MRI) performed prior to chemotherapy.

Consultation with thoracic surgeons suggested resection of the largest cystic lesion for pathology. Therefore, he underwent the third surgery under thoracoscope, and the largest cystic lesion in the right lung was resected.

### 2.6 Histopathology of the third surgery

**Figure 6:**
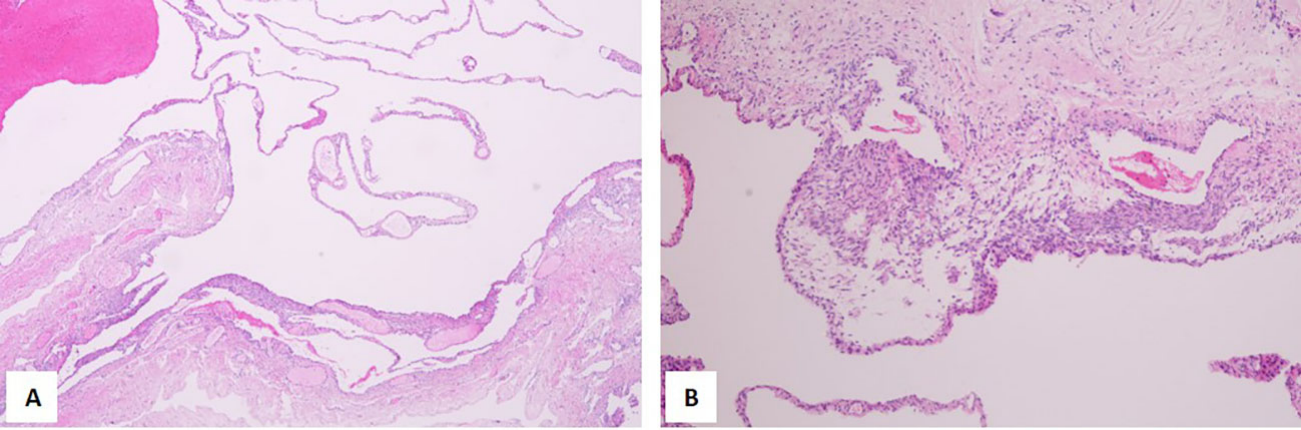
Hematoxylin–eosin (HE) staining of the lesion in the lung. **(A)** Magnification, ×4. **(B)** Magnification, ×10.

The lung tissue with lesion had a size of 3.2 cm × 2.3 cm × 1.5 cm, and a ruptured cyst was seen on the surface of the lung, with a diameter of 2.5 cm and wall thickness 0.1–0.2 cm. Macroscopic examination revealed the lesion as multilocular, with the majority of the lumen lined with a single cubic epithelium and a few lined with a pseudostratified ciliated columnar epithelium, under which focal or thin layers of dense oval or short fusiform cells were observed, which were arranged in nests or bundles (**Figure 6**). Immunohistochemistry yielded positive results for TTF1, CD31, SMA, catenin, and Masson and negative results for desmin, MyoD1, myogenin, S-100, EBER, and PAS. The Ki-67 index was 4%. A diagnosis of Ir type PPB was made.

### 2.7 Treatment after the third surgery

Chemotherapy was continued with the VAC regimen (vincristine, actinomycin D, and cyclophosphamide) for four cycles, during which the CT scan showed that the low-density nodules in the kidneys decreased in size and the cystic lesions in the right lung increased in size. Multidisciplinary team consultation did not suggest further surgery. Therefore, we continued vincristine and actinomycin D for three cycles, mainly aimed at nephroblastomatosis.

### 2.8 Molecular analysis

Genetic testing was performed on the renal tumor tissue and peripheral blood using the GenCap next-generation sequencing (NGS) method, which covers the exons and hotspot intronic regions of 890 genes and is capable of detecting multiple variant types, including gene mutations, copy number variations, and gene fusions. Molecular analysis identified three kinds of mutation in renal tumor tissue: *DICER1*: c.5113G>A p.E1705K (exon26); *DICER1*: c.4242dup p.E1415X (exon25) (**Supplementary Figure 1**); and *KRAS*: c.35G>A p.G12D (exon2). The variant frequencies were 5.63%, 5.62%, and 34.12%, respectively. The transcript used for *DICER1* was NM_030621, while that used for *KRAS* was NM_004985. The variant *DICER1*: c.5113G>A p.E1705K (exon 26) was not detected at a significant frequency in the gnomAD database of healthy individuals, but is curated in the ClinGen database and classified as a pathogenic variant. *DICER1*: c.4242dup p.E1415X (exon 25) was not found in the gnomAD database and was classified as a likely pathogenic variant according to the American College of Medical Genetics (ACMG) guidelines. *KRAS*: c.35G>A p.G12D (exon 2) is present in the gnomAD database at an extremely low allele frequency and is classified as a pathogenic variant in the ClinGen database. No germline mutation was detected.

Verification of the intestinal tumor and lung tumor both revealed *DICER1*: c.5113G>A p.E1705K (exon26), with variant frequencies of 87.55% and 21.72%, respectively, while *DICER1*: c.4242dup p.E1415X (exon25) was not detected (**Supplementary Figure 1**). The high variant frequency in the intestinal tumor indicated loss of heterozygosity of *DICER1*. No other pathogenic mutation was found in the lung tumor, while the intestinal tumor tissue was not sufficient for further tests. Therefore, we supposed it was the mosaic mutation model of *DICER1.*

### 2.9 Follow-up

The patient has been followed up for 27 months since the initial diagnosis. Imaging showed stable disease in both the kidneys and lungs, and the digestive tract was normal.

## 3 Literature review

By searching the literature in PubMed (from inception up to July 2024) using the term “*DICER1* mosaic mutation,” we found five articles reporting on 15 patients with mosaic *DICER1* mutation. Including our case, there were 10 patients with mosaic *DICER1* RNase IIIb mutations and six patients with mosaic *DICER1* LOF mutations. These mutations were detected in multiple primary neoplasms and/or non-neoplastic tissues (2–4) (**Tables 1**, **2**).

**Table 1:** Sequence results of mosaic *DICER1* RNase IIIb mutations.

First author	Patient	Tissue source	RNase IIIb domain hotspot mutation	Loss-of-function mutation
Brenneman (2)	1	Blood	c.5126A>G; p.D1709G	ND
		Normal lymph node		ND
Brenneman (2)	2	Blood	c.5125G>A; p.D1709N	ND
		Brain, PPB metastasis		Allele loss
Brenneman (2)	3	Blood	c.5125G>A; p.D1709N	ND
		Kidney, CN		c.1129G>A; p.V377I
		Lung, PPB type Ir		c.1200G>A; p.W400*
		Small intestine polyp		c.96G>A; p.W32*
Brenneman (2)	4	Blood	c.5428G>T; p.D1810Y	ND
		Normal fallopian tube		ND
		Lung, PPB type IR		ND
		Kidney, CN		c.1711delT; p.S571Vfs*16
		Ovary, SLCT		Allele loss
Brenneman (2)	5	Blood	c.5437G>C; p.E1813Q	ND
		Nasal cavity, NCMH		ND
		Thyroid, follicular Ca		Allele loss
		Ovary (right), SLCT		Allele loss
		Ovary (left), SLCT		c.4626delC; p.Q1542Hfs*18
Klein (3)	6	Blood	c.5138A>T; p.D1713V	ND
		Normal kidney		ND
		Wilms tumor		c.1304C>T; p.P453L
	7	Blood	c.5125G>T; p.D1709Y	ND
		Normal kidney		ND
		Wilms tumor		ND
De Kock (4)	8	Blood	c.5125G>C; p.D1709H	ND
		Pituitary blastoma		Allele loss
	9	Blood	ND	ND
		NCMH	c.5439G>C; p.E1813D	c.4651 4652insTGCT
		Normal right kidney		ND
		Normal right kidney		ND
Our case		Blood	ND	ND
		Wilms tumor	c.5113G>A; p.E1705K	c.4242dup; p.E1415X
		Small intestine polyp		NA
		PPB		NA

PPB, pleuropulmonary blastoma; CN, cystic nephroma; SLCT, Sertoli–Leydig cell tumor; NCMH, nasal chondromesenchymal hamartoma; ND, none detected; NA, not available.

* stands for a premature stop codon.

**Table 2:** Sequence results of mosaic *DICER1* loss-of-function mutations.

First author	Patient	Tissue source	Loss-of-function mutation	RNase IIIb domain hotspot mutation
Brenneman (2)	10	Saliva	c.1165-1174dupCCATATGAGC; p.R392Pfs3*	ND
		Fibroblasts		
		PPB type II		c.5439G>C; p.E1813D
Brenneman (2)	11	Blood	c.4426-4427insT; p.D1476Vfs5*	ND
		PPB type III		c.5424G>A; p.G1809R
Brenneman (2)	12	Blood	c.745C>T; p.Gln249*	ND
		PPB type I		c.5125C>T; p.D1709N
Brenneman (2)	13	Blood	c.2236A>G; p.R746G	ND
		PPB type III	NA	NA
Brenneman (2)	14	Blood	c.1716delT; p.F572Lfs*15	ND
		PPB type I	NA	NA
Bailey (5)	15	Blood	c.4754C>T; p.S1585	ND
		PPB type I	NA	c.5125C>T; p.D1709N

*PPB*, pleuropulmonary blastoma; *ND*, none detected; *NA*, not available.

* stands for a premature stop codon.

For the six patients with mosaic *DICER1* LOF mutations, the frequencies of mutations ranged from 1.1% to 17.2% of the allelic reads in DNA from the blood, saliva, or normal fibroblasts. All lesions were restricted to the lung, with three having a single focus of disease and the other three children each with two foci of disease (**Table 3**). Two of the six were deceased at a median age of 64.5 months (2, 3).

**Table 3:** Clinical features of patients with mosaic *DICER1* mutation.

First author	Patient	Type of mosaic mutation	Age at onset	SIP	IFMT	LC	PPB	CN	WT	NCMH	SLCT	TCa	TN	PinB	PitB	CBME	GS
Brenneman (2)	1	RNase IIIb	1–2 years	✓			✓	✓									
	2	RNase IIIb	0	✓			✓			✓							
	3	RNase IIIb	<1 year	✓			✓	✓									
	4	RNase IIIb	<1 year				✓	✓			✓		✓				
	5	RNase IIIb	1–2 years				✓	✓			✓	✓		✓		✓	
Klein (3)	6	RNase IIIb	9 months			✓			✓								✓
	7	RNase IIIb	14 months			✓			✓								✓
De Kock (4)	8	RNase IIIb	NA			✓		✓							✓		
	9	RNase IIIb	10 days	✓		✓		✓		✓							
Our patient		RNase IIIb	6 months	✓	✓		✓	✓	✓								
Brenneman (2)	10	Loss of function	NA				✓										
	11	Loss of function	NA				✓										
	12	Loss of function	NA				✓										
	13	Loss of function	NA				✓										
	14	Loss of function	NA				✓										
Bailey (5)	15	Loss of function	22 months				✓										

*SIP*, small intestinal polyp(s); *IFMT*, intestinal fusiform mesenchymal tumor; *LC*, lung cysts; *PPB*, pleuropulmonary blastoma; *CN*, cystic nephroma; *WT*, Wilms tumor; *NCMH*, nasal chondromesenchymal hamartoma; *SLCT*, Sertoli–Leydig cell tumor (ovary); *TCa*, thyroid carcinoma; *TN*, thyroid nodule(s); *PinB*, pineoblastoma; *CBME*, ciliary body medulloepithelioma (eye); *GS*, GLOW syndrome.

For the 10 patients with mosaic *DICER1* RNase IIIb mutations, the frequencies of mutations ranged from 0.04% to 35% of the allelic reads in DNA from the blood, normal lymph node, or normal kidney. All presented with multiple cysts of the lungs and/or kidneys, with the first one appearing within 15 months of birth, which were accompanied or followed in all cases by multiple *DICER1* syndromic tumors (**Table 3**). All were alive during the follow-up (2, 4).

## 4 Discussion

Studies have revealed that predisposing germline *DICER1* pathogenic variants are predominantly LOF variants. LOF mutations are widely distributed in the whole gene, and the most common mutation is the truncated mutation, such as deletion, repetition, insertion, conversion, translocation, or nonsense mutation of the gene sequence (6). In addition, *DICER1*-related tumors very characteristically harbor an additional RNase IIIb hotspot mutation. RNase IIIb hotspot mutations are missense alterations involving one of six codons within the sequence encoding the *DICER1* RNase IIIb domain (7).

*DICER1*-related tumor predisposition is a well-known cancer-predisposing disorder caused by pathogenic variants in the *DICER1* gene, which are associated with lifetime risks of a variety of neoplastic and dysplastic lesions, such as PPB, cystic nephroma (CN), ovarian Sertoli–Leydig cell tumor (SLCT), multinodular goiter, cervix embryonal rhabdomyosarcoma, Wilms tumor, nasal chondromesenchymal hamartoma, ciliary body medulloepithelioma, differentiated thyroid carcinoma, pituitary blastoma, pineoblastoma, and sarcomas of different sites including the uterine cervix, kidney, and brain (6). These neoplasms are believed to evolve via an unusual form of Knudson’s two-hit hypothesis (8).

Research has revealed three types of “two-hit” tumorigenesis: i) LOF mutation is the most common form, i.e., one *DICER1* allele has a LOF mutation, while the other allele has a hotspot mutation in the RNase IIIb domain; ii) tumor-specific, biallelic *DICER1* mutations, which account for approximately 10% of PPB cases; and iii) in particular, approximately 10% of predisposing *DICER1* mutations are mosaic rather than germline. Mosaic mutation presents in some but not all cells of the body as it occurs during the post-zygotic embryonic development rather than being present in the zygote. Brenneman et al. categorize a *DICER1* mutation as mosaic when the following criteria are met: i) The mutation is evidently not a germline allele because it is present at sub-heterozygous frequency (arbitrarily taken as below 35% of reads) in the peripheral blood and/or other normal tissue samples, and ii) the mutation is evidently not specific to a tumor because the same mutant allele is detected in one or more normal, non-neoplastic tissue samples or the same mutant allele is detected in multiple primary tumors arising in different organs (2). In our case, *DICER1* c.5113G>A was not specific in a certain type of tumor, nor germline; therefore, we consider it as a mosaic mutation. However, we did not perform further sequencing to determine *DICER1* c.5113G>A in other tissues. The intestinal tumor tissue was not sufficient for further tests, but appears to be a novel lesion that needs further workup for characterization.

In the mosaic mutation case series, all were diagnosed with *DICER1*-related tumor predisposition early and presented with multiple cysts of the lungs and/or kidneys, which were accompanied or followed by multiple *DICER1* syndromic tumors. Our case also matched the recently discovered new feature of mosaic RNase IIIb hotspot mutations—relative to juvenile-type intestinal polyps and intussusception (2). Moreover, the patient has intestinal fusiform mesenchymal tumor, suggesting the possibility of more tumors related to the mutation. Although the reports of intestinal polyps in the *DICER1* setting are not detailed in terms of their pathological features, it appears that they are more aligned with hamartomatous types, including juvenile polyps/juvenile polyposis syndrome, P-J polyps of P-J syndrome, and hamartomatous polyps in the *PTEN* hamartoma tumor syndrome (1). Although all of the patients with mosaic hotspot mutations were alive during the follow-up, their clinical experiences have been complicated and arduous.

Patients with mosaic *DICER1* RNase IIIb hotspot mutations occasionally manifest global developmental delay and overgrowth, considered by some authors as a distinct entity called global developmental delay, lung cysts, overgrowth, and Wilms tumor (GLOW) syndrome (3). Our case did not appear to have developmental delay or overgrowth. In the report of Salvador et al., only 3 out of 16 patients with constitutional and postzygotic forms of pathogenic *DICER1* RNase III missense variants developed the complete GLOW syndrome and 10 out of 16 were not diagnosed with any GLOW manifestations, suggesting that the GLOW phenotype lacks specificity to pathogenic RNase III missense variants (9).

For the treatment of the patient, the P-J polyps and low-grade fusiform mesenchymal tumor were resected and did not need further treatment. The nodules in the kidney were getting smaller after chemotherapy and the lesions were diffused; hence, there was no need for surgery. Regarding the cystic lesions in the lung, they were also diffused and cannot be totally resected. Considering the pathology being Ir type PPB, we administered four cycles of the VAC regimen, as well as a VA regimen afterward that was mainly aimed at nephroblastomatosis. Considering that chemotherapy may increase the risk of other malignant diseases in a patient with *DICER1*-related tumor predisposition and that the lesions were stable, we then stopped the treatment and observed.

Due to the high risk of surviving other tumors in the future, similar to other patients with *DICER1*-related tumor predisposition, our patient needed standard surveillance during and after treatment, not only for the sites of previous lesions but also for other sites with high risk of cancer, including the thyroid, the central nervous system, and the head and neck region, following previously published recommendations (10).

## Data Availability

The original contributions presented in the study are included in the article/**Supplementary Material**. Further inquiries can be directed to the corresponding author.
