# Supplementary material for: Mosaic DICER1 RNase IIIb hotspot mutation with multiple tumors: case report and literature review
Source: Front Oncol. 2026 Apr 27;16:1777961. doi: 10.3389/fonc.2026.1777961 (PMC13158117; doi:10.3389/fonc.2026.1777961)
Supplement: Supplementary file 1 [file DataSheet1.docx]

**Supplementary Figures**

**Supplementary Figure 1-A**


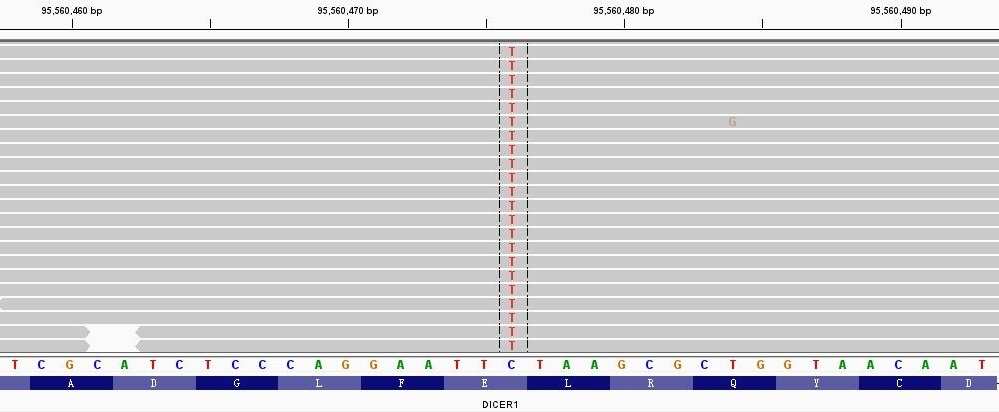


**Supplementary Figure 1-B**


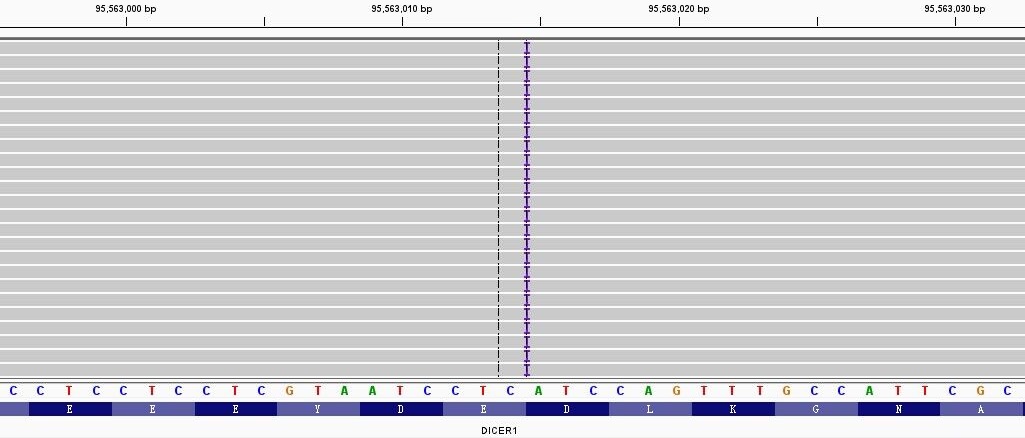


**Supplementary Figure 1-C**


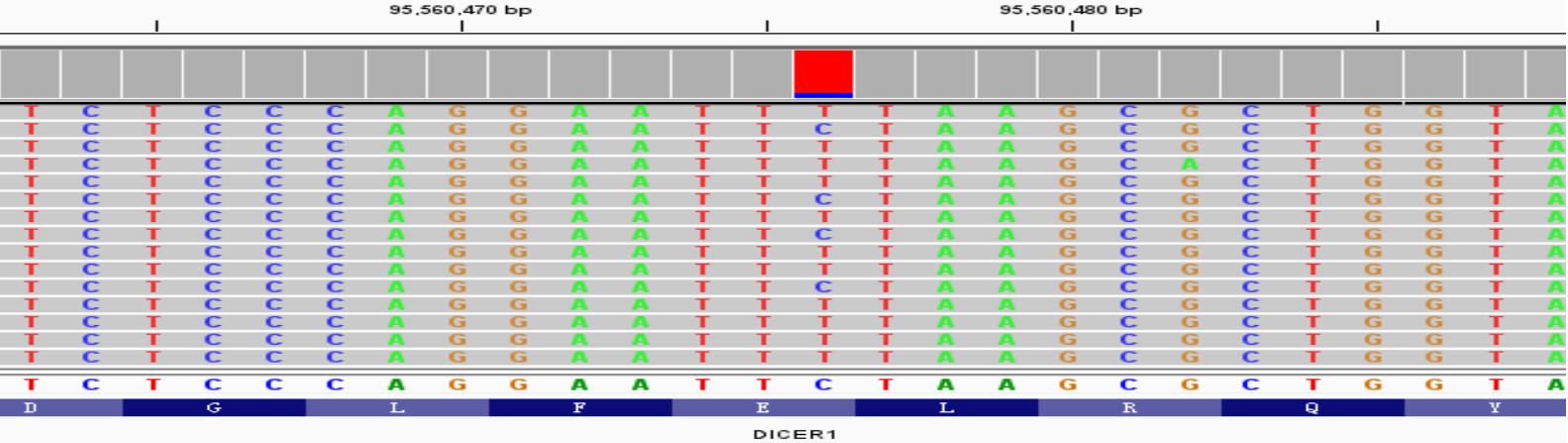


**Supplementary Figure 1-D**


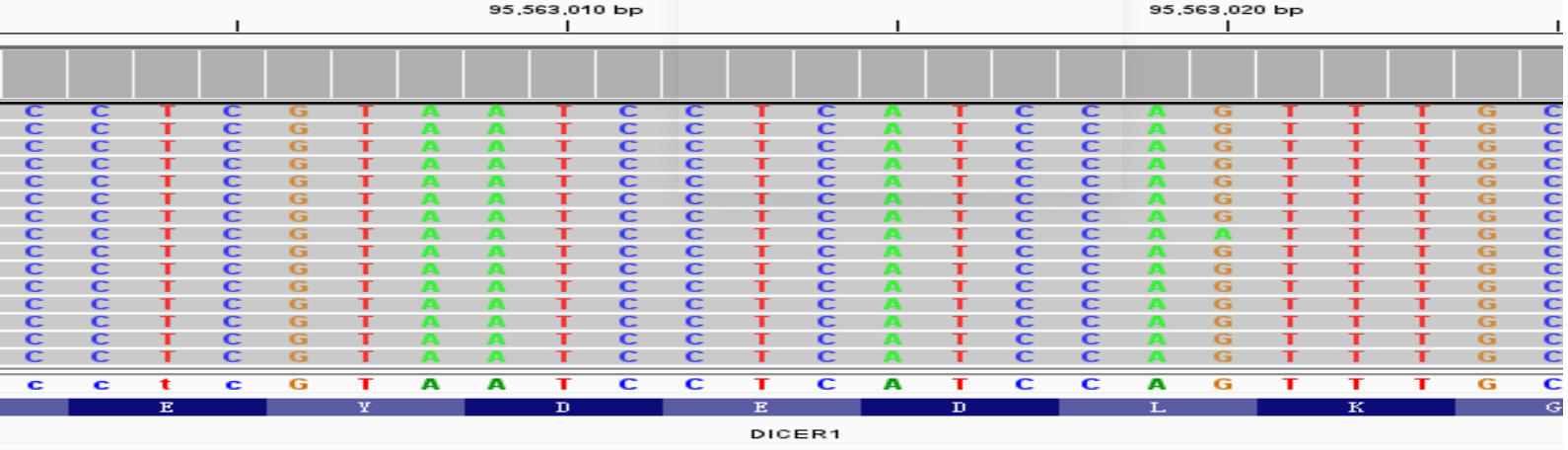


**Supplementary Figure 1-E**


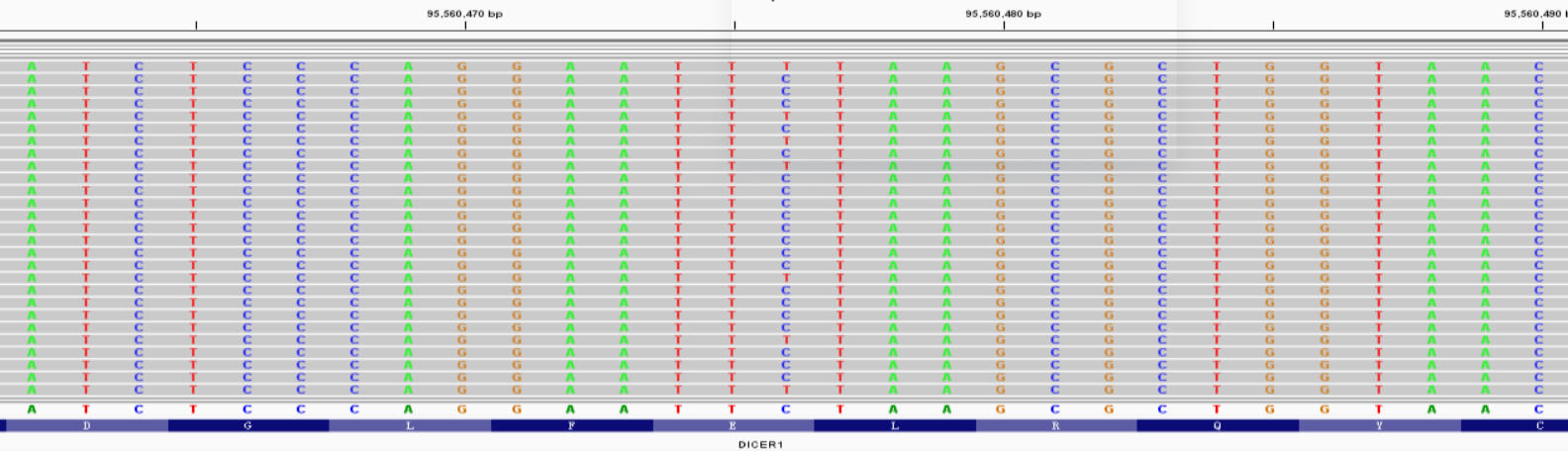


**Supplementary Figure 1-F**


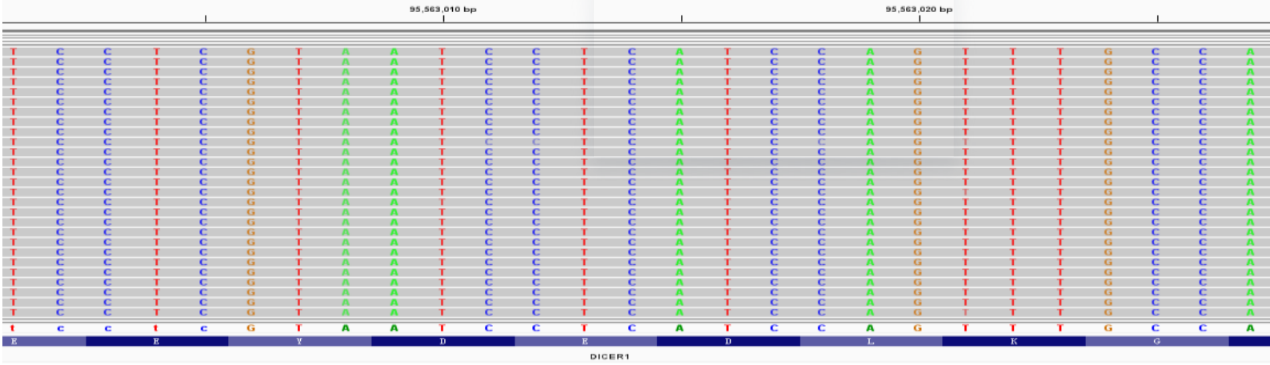


**Supplementary Figure 1.** Bam figures shows DICER1 exon26 c.5113G>A p.E1705K(A) and DICER1 exon25 c.4242dup p.E1415X(B) were detected in the kidney tumor. DICER1 exon26 c.5113G>A p.E1705K was detected both in intestinal tumor(C) and lung tumor(E), while DICER1 exon25 c.4242dup p.E1415X was not detected either in intestinal tumor(D) and lung tumor(F).
